# Regulation of COVID-19 fake news infodemic in China and India

**DOI:** 10.1177/1329878X20948202

**Published:** 2020-11

**Authors:** Usha M Rodrigues, Jian Xu

**Affiliations:** Deakin University, Australia

**Keywords:** China, COVID-19, fake news, free speech, government regulation, India, social media

## Abstract

During the recent outbreak of coronavirus, the concern about proliferation of misleading information, rumours and myths has caused governments across the world to institute various interventionist steps to stem their flow. Each government has had to balance the dichotomy between freedom of expression and people’s right to be safe from the adverse impact of inaccurate information. Governments across the world have implemented a number of strategies to manage COVID-19 including issuing public advisories, advertising campaigns, holding press conferences and instituting punitive regulations to combat the distribution of false and misleading information. We examine the two most populous countries’ governments’ response to the scourge of fake news during COVID-19. China and India are the most challenging nations to govern in terms of their sheer size and diversity of their population. Each country’s government has taken several steps to minimise the impact of fake news during COVID, within its own political system.

## Introduction

The death toll from COVID-19 has reached 5.5 million mark, while nearly 12 million people have been infected with the pandemic (Coronavirus.jhu.edu, 2020). In addition to informing citizens about how to minimise the risk of novel coronavirus, governments and health authorities across the world have been busy debunking false and misleading information distributed on private and public social networks in relation to COVID-19 (UNDP.org, 2020). Researchers at the Bruno Kessler Foundation recently analysed 112 million public social posts related to the pandemic and found that 40% came from unreliable sources, while 42% were circulated by bots (UNDP.org, 2020). The United Nations Development Programme has called on governments across the world to ‘step up to lead the fight against a growing tide of false, inflammatory and misleading information that threatens to worsen the already severe impacts of the virus’ (UNDP.org, 2020). We examine the two most populous nations, Chinese and Indian governments’ interventionist steps to stem the flow of fake news, particularly on social media platforms since late January 2020. Despite early successes, China is still managing the second wave of COVID-19 outbreak, whereas India is yet to go past the first stage of coronavirus infections, increasing the significance of the two countries response to the infodemic of fake news.

## Local conditions drive the government regulation agenda

Social media sites and apps with their capacity to allow many-to-many communication and the ease of sharing of micro-content have become an important avenue for exchange of news and views in recent times. However, the proliferation of fake news, the concerns about data privacy and the commercialisation of user data have ensured that the debate about government regulation of social media platforms continues. In the past, Facebook CEO Mark Zuckerberg has defended the open platform of Facebook as a place for freedom of speech (Paul, 2019), while Twitter has in recent times labelled the US President Donald Trump’s tweet as ‘manipulated media’ (Zakrzewski, 2020). Social media companies are coming under increased pressure to moderate misleading and false content being shared on their platforms. Scholar have noted the changing discourse about social media being a tool for democratic uprising (Lewis and Molyneux, 2018) and activism (Xu, 2016) to being a means for government surveillance, corporate data collections (Vaidhyanathan, 2018), political manipulation (Gunther et al., 2018; Rodrigues, 2019) and a space for radicalisation (Figoureux and Van Gorp, 2020).

Governments in more liberal and democratic countries, with higher level of media and informational literacy, remain torn between the need to preserve citizens’ freedom of speech and curtail the circulation of fake news (Rochefort, 2020). However, governments in authoritarian governments are less concerned with the accusation of overstepping the boundaries of free speech (Tkacheva et al., 2013). Within this scenario, each government is responding to the scourge of fake news within their own political and media systems. Some governments remain at arm’s length by working with social media companies where they are asked to label political advertising and remove misleading and false posts, while other governments appoint a taskforce to deal with fake news and sponsor media literacy campaigns (Funke and Flamini, 2019). A number of governments have proposed or passed laws that criminalise creating and spreading of rumours on social media sites and apps, while others have passed specific laws against hate speech on social platforms. These laws have raised civil libertarians’ concerns about the abuse of such regulations by authorities and imprisoning of journalists and detractors under the guise of regulating fake news on social media (Berger, 2019). Meanwhile, the Indian government stands out for its extreme practice of turning off the Internet to quell the spread of rumours on WhatsApp (Nazmi, 2019). The official rationale for the Internet shutdowns, which has affected the troubled region of Kashmir more than any other parts of India, is to combat fake news, hate speech and secure public safety and national security. However, critics say that the main aims of the Internet shutdowns is to minimise political instability, communal violence, civil protests and controlling information during elections, according to a #KeepItOn report on digital rights of users (AccessNow 2019).

## Communication infrastructure in China and India

Chinese media, which are highly market-oriented but Communist Party-controlled, include three leading state media (China Central Television, Xinhua News Agency, *People’s Daily*), two most popular social media sites (Weibo and WeChat) and a search engine (Baidu). Without having access to the world’s most popular news sharing platforms, such as YouTube, Facebook, WhatsApp and Twitter, most Chinese people receive news information from traditional state media and the censored ‘intranet’ including commercial news portals, audio-visual sites and social media sites. China has the world’s largest Internet population, reaching 904 million with the Internet penetration of 64.5% by March 2020 (CNNIC, 2020). There are 731 million online news consumers in China, with 726 million accessing news on their mobile devices (CNNIC, 2020). Weibo with 550 million and WeChat with 983 million users dominate the social media landscape in China (Statista.com, 2020b). The two mobile platforms as main sources of news for Chinese users are the focus of the state’s cyber governance during the coronavirus crisis period. Due to China’s strong control and complicated censorship of the Internet and social media, it is ranked 177th out of 180 countries in the World Press Freedom index (RSF.org, 2020).

About 86% of 1.38 billion Indians subscribed to a mobile phone at the end of January 2020 (TRAI.gov.in, 2020). An overwhelming majority access the wireless service via private providers, while about 10% of mobile users subscribe to a public service provider. Most of the Internet users (687 million) access the Internet and news on their mobiles (Statista.com, 2020a). With over 400 million monthly active users, the social networking site Facebook dominates the Indian market, while WhatsApp is the most popular messenger application (Datareportal.com, 2020). Meanwhile, India also has one of the largest traditional media with over 100,000 news publications, about 850 television and 1000 radio channels. The media derives its freedom, to question those in power, from the Constitution of India’s article 19, which guarantees all its citizens the fundamental right of ‘Freedom of Speech and Expression’, with a few caveats such as free speech cannot be used to promote communal disharmony, go against public decency, and compromise security and national sovereignty. However, on the 2020 World Press Freedom index, India dropped to 142nd ranking because of recent attacks on journalists by pro-government political activists and the imposition of electronic and the Internet blackouts in the Kashmir Valley (RSF.org, 2020).

## Government response to COVID-19 misinformation

In China, the synonymous term for ‘fake news’ is ‘rumours’. Since President Xi Jinping came to power in late 2012, the Chinese government has launched a series of campaigns to combat online rumours (Benny and Xu, 2018). Under the leadership of the Cyberspace Administration of China (CAC), Chinese Internet giants, including Weibo, WeChat, Baidu and Toutiao, joined forces against online rumours and have developed a rumour-reporting and refutation mechanism. In August 2018, the Illegal and Unhealthy Information Reporting Centre, affiliated to CAC, established a national-level rumour-refuting platform (http://www.piyao.org.cn/). The platform has integrated over 40 local rumour-refuting platforms and uses artificial intelligence to identify misinformation (Qiu and Woo, 2018). China’s first Cybersecurity Law was implemented in June 2017 and various regulations on Internet group information, online comment and live-streaming services have provided legal and regulatory grounds for the governance of online rumours (Xinhuanet.com, 2019). China’s well-established ‘anti-online-rumour’ mechanism as well as its advanced and sophisticated Internet censorship have made the Chinese government fully prepared for the campaign against fake news in coronavirus. In another word, COVID-19 is a critical moment for the government to test the capability of its ‘anti-online-rumour’ system and mechanism.

The Chinese government has taken a number of ‘anti-online-rumour’ measures during COVID-19. The national and local rumour-refuting platforms have set-up dedicated rumour-refuting sections. For example, the local rumour-refuting platforms in Beijing had exposed 600 rumours about COVID-19 by 24 May and also clarified corresponding truth for all misinformation (Qianlong.com, 2020). Since late January 2020, popular social media platforms, such as WeChat and Douyin, have initiated special rectification campaigns against COVID-19-related rumours. Fake news has been swiftly removed and accounts that spread misinformation shut down (Chinanews.com, 2020). Major news apps such as Xinhua News, Tencent News and WeChat public accounts of the Communist Youth League Central Committee as well as Sina Weibo provide dedicated sections for rumour detection and fact checking. Various local governments, with the support of propaganda department, health commission and police bureau, have initiated anti-rumour campaign to combat locally relevant fake news. Rumour-mongers, who spread fake news on social media, have been detained by local police in accordance with the Security Administration Punishment Law (Sina.com, 2020). The news of punishing rumour-mongers has been widely circulated on social media to deter others. Propaganda banners, ‘Do not produce, circulate or believe rumors and be law-abiding citizens’, have been plastered all over the cities and villages. Last but not the least, the central and various local governments along with state media proactively use social media to feed the public with timely and authoritative official news information.

The Indian Prime Minister Narendra Modi’s government launched the Digital India initiative in 2014 after coming to power. The arrival of affordable mobile service has witnessed a dramatic growth in the number of people accessing the Internet and social media both in urban and rural India (Rodrigues and Niemann, 2019). A significant proportion of sharing of messages happens on WhatsApp and other encrypted messenger apps, making it impossible to detect the source and nature of messages being shared. Following the arrival of COVID-19, the Indian government invoked the Disaster Management Act 2005 to impose a nationwide 3-week lockdown and made it a criminal offence to create ‘panic’ in India. The government also referenced the colonial-era Epidemic Diseases Act 1897 to make a misleading publication punishable. On 20 March, the Ministry of Electronics and Information Technology issued an advisory to social media companies, Facebook, Twitter, ShareChat and WhatsApp, ‘to disable or remove’ any false news spreading from their platforms on a priority basis and initiate user ‘awareness campaign’ to stop them from circulating any false or misinformation concerning coronavirus which is likely ‘to create panic among the public’ (PTI, 2020).

On 21 March, the Indian government set up a ‘Mygov Corona’ chatbot on WhatsApp to provide coronavirus-related information to the users (Singh, 2020). The bot is built by a private telecom giant, while the information is provided by the Ministry of Health. Six hours before announcing the nation-wide lockdown, the Indian Prime Minister personally asked 20 news media owners and editors to publish positive stories about COVID-19. In a disappointing move for mainstream news media, the Indian government also approached the apex judicial body in the country, the Supreme Court, to refrain news outlets from publishing any COVID-19-related news without clearance from the government on the grounds that ‘fake or inaccurate’ reporting could cause panic in the country. The Supreme Court denied the request, but directed news outlets to use official version of the COVID-19 developments in their reporting. This judicial direction caused concern among journalists, who in the past have not been forced to use official information in their news reports (CPJ.org, 2020). However, it is worth noting that some of the unverified myths and cures such as cow urine could combat the coronavirus have been spread by some of the politicians and celebrities on social media platforms (Mohan, 2020). The Indian government has continued the strategy of shutting down the Internet in parts of India to curb the flow of fake and hate messages, a strategy praised by the Chinese state media (Chilappa, 2020).

## Discussion and analysis

China, with its authoritarian political system and stricter information control, has effectively restricted the circulation of fake news/rumours during COVID-19 outbreak. The authoritarian regime put aside the ‘freedom of expression’ ethos during the crisis period and emphasised ‘social responsibility’, ‘public security’ and ‘social order’ as rationale for censoring the information on social media platforms. These reasons are well publicised to help establish an image of a government that is effective and responsible during a crisis. Whereas India as a democracy has had a mixed and chaotic track record in combatting fake news. Although the government has continuously worked with big technology companies to restrict the spread of fake news, some of its politicians have continued to undermine the government’s effectiveness by spreading unproven and inaccurate health information (Mohan, 2020). Some argue that the Indian government crossed the line to *authoritarianism* when it asked the Supreme Court to direct the mainstream news media to not publish any COVID-19-related news without clearance (CPJ.org, 2020). However, the application was declined by the apex court, upholding Indian citizens’ Constitutional right of ‘freedom of speech’. However, the Indian central and state governments have continued the strategy of the Internet shutdown in various parts of the country during COVID-19 to control the flow of information, which some argue is more to do with controlling political opposition and civil protests (Nazmi, 2019). This also means that the widely criticised Internet censorship and shutdown in authoritarian states is also likely to be utilised by liberal and democratic nations in special circumstances. The circulation of fake news, rumours and misinformation during COVID-19 has pushed each government to navigate the complicated trade-off between state power and freedom of speech of its citizens. The Chinese and Indian governments’ response for combating fake news during a significant health crisis provides a sketch of how different political systems are used to manage the complex relationship with their citizens.
